# Risk Factors Associated with Recurrent *Clostridioides difficile* Infection

**DOI:** 10.3390/healthcare8030352

**Published:** 2020-09-21

**Authors:** Nicoleta Negrut, Simona Bungau, Tapan Behl, Shamim Ahmad Khan, Cosmin Mihai Vesa, Cristiana Bustea, Delia Carmen Nistor-Cseppento, Marius Rus, Flavia-Maria Pavel, Delia Mirela Tit

**Affiliations:** 1 Department of Psycho-Neuroscience and Recovery, Faculty of Medicine and Pharmacy, University of Oradea, 410028 Oradea, Romania; lnm_n10@yahoo.com (N.N.); delia_cseppento@yahoo.com (D.C.N.-C.); 2 Department of Pharmacy, Faculty of Medicine and Pharmacy, University of Oradea, 29 N. Jiga St., 410028 Oradea, Romania; mirela_tit@yahoo.com; 3 Department of Pharmacology, Chitkara College of Pharmacy, Chitkara University, Punjab 140401, India; 4 Faculty of Medicine and Pharmacy, University of Oradea, 410073 Oradea, Romania; doc5984@gmail.com; 5 Department of Preclinical Disciplines, Faculty of Medicine and Pharmacy, University of Oradea, 410073 Oradea, Romania; v_cosmin_15@yahoo.com (C.M.V.); cristianabustea@yahoo.com (C.B.); flavia.bontze@gmail.com (F.-M.P.); 6 Department of Medical Disciplines, Faculty of Medicine and Pharmacy, University of Oradea, 410073 Oradea, Romania; rusmariusr@yahoo.com

**Keywords:** *Clostridioides difficile*, recurrent disease, risk factors, antibiotic-associated diarrhea, ATLAS score, Charlson Comorbidity Index

## Abstract

*Clostridioides difficile* (CD) is responsible for nosocomial diarrhea syndrome with possible severe progression. Recurrence of the disease induces higher health system costs, as well as exposes patients to additional health risks. Patients with recurrence of this disease are difficult to identify, so the purpose of this study is to quantify various demographic, clinical, and treatment factors that could prevent further progression to recurrence of the disease. In the period 2018–2019, about 195 patients were diagnosed with more than one episode of CDI in the three months following the first episode. The recurrence rate for CDI was 53.84% (60.95% for one episode and 39.05% for multiple episodes). Most commonly afflicted were 60–69-year-old patients, or those with higher Charlson Comorbidity Index (CCI). Multiple analyses associated cardiovascular (odds ratios (OR) = 3.02, 95% confidence intervals (CI) = 1.23–7.39, *p* = 0.015), digestive (OR = 3.58, 95% CI = 1.01–12.63, *p* = 0.047), dementia (OR = 3.26, 95% CI = 1.26–8.41, *p* = 0.014), immunosuppressive (OR = 3.88, 95% CI = 1.34–11.21, *p* = 0.012) comorbidities with recurrences. Risk factor identification in the first episode of CDI could lead to the implementation of treatment strategies to improve the patients’ quality of life affected by this disease.

## 1. Introduction

Patients with *Clostridioides difficile* infection (CDI) face high treatment costs in addition to a high risk of mortality. Furthermore, there is a risk of recurrence within the 90 days following the initial episode, being accurately treated. Most CDI patients return to their social and family lives after clinical remission and completion of treatment. A patient with recurrence within the three months following the initial episode is, obviously, a potential source of infection for the community. A new episode of CDI in a patient can lead to increased mortality risk, isolation problems, additional costs, treatment dilemmas, as well as the risk of decompensation of pre-existing comorbidities. Furthermore, the risk for new diarrhea episodes increases from 25 to 65% after the first recurrence episode [1]. Each new episode raises the risk of a future episode by about 20% [2].

So far, the exact causes of the recurrences are unknown. However, the persistence of the sporulated forms in the patient’s microbiota or the re-exposure of patients with dysbiosis to the contaminated environment can be some of the causes that may lead to recurrences. It is difficult to predict the risk of recurrence. The reemergence of the clinical picture, often more severe, after proper treatments for CDI, has led to sustained research efforts to identify the factors that could predict the recurrences.

In a study conducted by Daniela Knafl et al. on 144 patients diagnosed with CDI (who presented at the University Hospital of the Medical University of Vienna in 2012), it was revealed that serum albumin could be used as a predictor for recurrence of CDI in the 90 days following the first episode [3].

Abhishek Deshpande et al., in a meta-analysis involving 33 studies (18,530 patients), concluded that in patients above 65 years old—antibiotics or proton-pump inhibitors used after the first episode of CDI, renal failure, and previous use of fluoroquinolones are risk factors for recurrence of CDI [4]. However, the medical literature is contradictory. Thus, Krishna Rao et al., in a study of 927 patients from Michigan, USA, claims that only ribotype 027 increases the risk of recurrence, whereas age, proton pump inhibitors, or antibiotics do not [5].

Actual medical literature has shown that therapy for the first episode of CDI may lower the risk of recurrence. According to medical guidelines, the etiologic treatment of CDI could be done with metronidazole, vancomycin, or fidaxomicin. Until now, it was known that fidaxomicin induces a lower recurrence rate compared with vancomycin in the cases of patients infected with non–North American Pulsed Field type 1 strains of *Clostridioides difficile* (CD) [6,7,8]. Fidaxomicin is a well-tolerated oral macrocyclic antibiotic with a narrow spectrum of activity. It lowers Bacteroides species (belonging to the habitual microbiota) without reported resistance (until now); however, it is inaccessibly priced for most patients. Another new option to reduce the recurrence of CDI is a fully humanized anti-CD toxin B monoclonal antibody (bezlotoxumab) [9]. It targets the CD toxin and does not disturb the patient’s microbiota. To date, the drug is in a clinical trial, phase 4 (ClinicalTrials.gov Identifier: NCT03937999), but for now, accessibility to it is low [10]. Complementary therapy with essential oils or fruit extracts were evaluated; the results, so far, being favorable regarding the diminishing of various pathogen multiplication (including of CD) [11,12,13,14].

Therapeutic options in patients with relapses are limited and often expensive, which requires identification of patients at risk of relapse from the first CDI episode.

The present study aims to identify patients at risk of recurrence, taking into account the existence of comorbidities, demographic indicators, and treatment with symbiotics used after the episode of CDI.

## 2. Materials and Methods

A two-year retrospective study (from 1 January 2018, to 31 December 2019) was performed at the Department of Infectious Disease of “Gavril Curteanu” Municipal Hospital, Oradea, Bihor County, Romania. All patients (with diarrheal syndrome) admitted to the department were analyzed for CDI. Demographic data (age, gender, residence), past medical history, as well as the ATLAS score (it is a score having five components, as follows: age, treatment with systemic antibiotics, leukocyte, albumin and serum creatinine—measuring the renal function) count, for severity of the positive cases, were analyzed. Fever, treatment with systemic antibiotics during CDI, leukocyte count, serum albumin level, and serum creatinine level were observed for the ATLAS score [15]. The ATLAS score is used to predict mortality at 30 days by CDI and to stratify the severity of the infection at the time of diagnosis. It is based on the 6 components described above, each being evaluated with a score between 0 and 2 points. The sum of the points varies from 0 to 12. The score from 0 to 3 is considered mild CDI, from 4 to 6 as moderate disease, and from 7 points as a severe episode of CDI.

The following past medical histories were followed: cardiovascular diseases, myocardial infarction, congestive heart failure, peripheral vascular disease, cerebrovascular accident, transient ischemic attack, diabetes mellitus, neoplasm (solid tumor, leukemia, lymphoma), chronic kidney disease, liver disease (cirrhosis, chronic hepatitis), chronic obstructive pulmonary diseases, psychiatric diseases, dementia, hemiplegia or paraplegia, peptic ulcer disease, connective tissue disease, and acquired immunodeficiency syndrome. Age associated with past medical history described above was used for the Charlson Comorbidity Index (CCI) [16]. The CCI is currently used to calculate 10-year survival, based on 17 associated comorbidities associated with mortality. Each disease is assigned a score ranging from 0 to 6 points. The sum of the points varies from 0 (no disease burden) to 29 (maximal disease burden).

The identification of the CD toxin was the inclusion criterion for patients in the study. All of the patients included in the study reported their first episode of diarrhea due to CDI at the moment of inclusion. No patient received symbiotic drugs one month before the onset of the CDI episode. Patients who were diagnosed with recurrent CDI (R) at the first medical visit were excluded from the study. Recurrence of CDI was considered in the case of the patients with demonstrated CDI in the 3 months after the correctly treated first episode of CDI. Non-recurrent (NR) infection was considered in the case of patients who did not have CDI in the same period. All patients were treated with metronidazole and vancomycin, according to the national methodology for CDI, valid for the studied years. Some patients with CDI, after the initial episode of CDI, followed a 3-month treatment with symbiotic drugs containing *Streptococcus faecalis* T-110—180 million colony-forming units (CFU), *Clostridioides butyricum* TO-A—12 million CFU, *Bacillus mesentericus* TO-A—6 million CFU, *Lactobacillus sporogenes*—300 million CFU daily. Because of a lower number of cases, patients treated with fidaxomicin were excluded from the current study.

### 2.1. Ethical Statement

The study complied with the World Medical Association Code of Ethics (2019) and had the approval of the Ethics Commission of the Medicine and Pharmacy Faculty, University of Oradea, number 1/05.26.2020. At hospital admission, each patient signed an informed-consent document.

### 2.2. Diagnosis of CDI

Patients with more than 3 unformed stools (Bristol scale 5–7) daily were checked for fecal A, B, or binary toxin (BT) by chromatographic immunoassay, or real-time polymerase chain reaction (RT-PCR). RT-PCR procedure does not determine sporulated forms.

The chromatographic immunoassay available in the clinic, in the time period followed was CerTest CD glutamate dehydrogenase (GDH)+ toxin A + B, (CerTest Biotec, Spain). Sensitivity for toxin A was 96.6% (95% confidence interval (CI), 92.2 to 99.9%), for toxin B, it was 100% (95% CI, 92.2 to 99.9%), specificity for toxin A was 100% (95% CI, 96.2 to 100%), and for toxin B, 98.9% (95% CI, 94 to 100%) [17].

The negative cases at the chromatographic immunoassay, with a clinical picture suggested for CDI, were checked for the presence of BT in a private laboratory, using Cepheid Xpert CD BT Assay (Cepheid, Sweden). The sensitivity of the test for identifying CD toxin gene B and BT gene was 93.39%, and the specificity 94.02%, respectively [18].

The stool samples were collected in an empty sterile container, transported at 5 °C to the laboratories, and analyzed in maximum of 2 h after the samples were collected.

### 2.3. Statistical Analysis

The statistical analysis was generated using Statistical Package for the Social Sciences, version 26. Odds ratios (OR) and 95% CI were calculated for quantification of the strongest associations. The calculation of *p* values was performed using Student’s t-test, chi-squared test, and logistic regression analysis. The statistical significance was considered for *p* values as being <0.05.

## 3. Results

A total of 202 patients were declared eligible during the two years of study. Five cases could not be followed up for three months and two cases were treated with fidaxomicin. A total of 195 patients remained in the study. The lot was relevant for a 95% probability. The recurrence rate for CDI was 53.84% (105 in the R group and 90 in the NR). From group R, 64 (60.95%) patients had a single recurrence for CDI, and 41 (39.05%) cases had multiple episodes of infection in the follow-up period.

**Figure 1:**
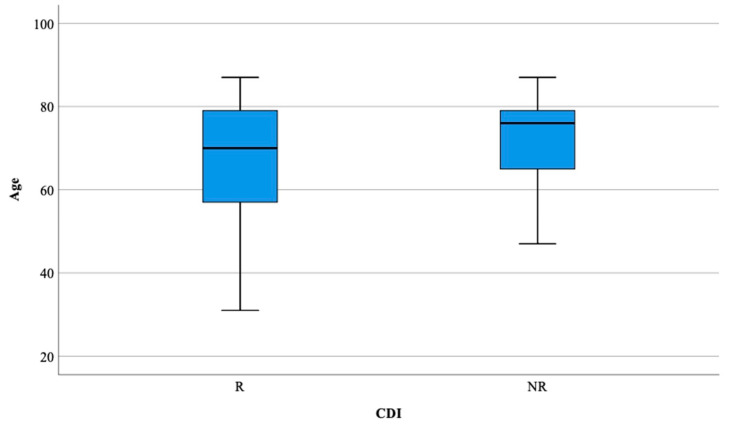
Distribution of the patients according to ages in both groups. R—recurrent; NR—non-recurrent.

The mean age of the R group did not differ statistically from the NR group (68.67 ± 14.84 vs. 69.6 ± 15.24, *p* > 0.05) (Figure 1). Patients aged 60–69 were, statistically, significantly more exposed to CDI recurrence (Table 1).

**Figure 2:**
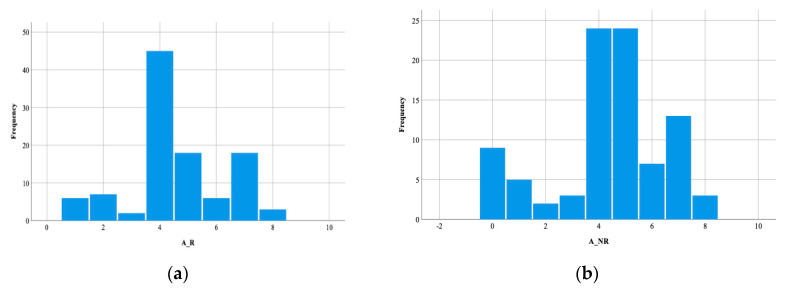
Histogram of ATLAS (age, treatment with systemic antibiotics, leukocyte, albumin and serum creatinine) values: (**a**) in the recurrent group; (**b**) in the non-recurrent group. A_R—ATLAS values in the recurrent group; A_NR– ATLAS values in the non-recurrent group.

The population from the urban area had 40.95% (43) recurrences of CDI, and those from the rural area, 67.77% (61) had no recurrences. The difference was not statistically significant. The male distribution did not differ significantly between the two groups (R group—69, 65.71% cases, NR group—30, 33.33%; *p* > 0.05). The number of patients treated initially with metronidazole did not differ significantly in the two groups (R group—40, 38.09% cases, NR group—35, 38.89%; *p* > 0.05). The analysis of the ATLAS values of the patients with CDI, during the studied period, revealed a value of 4.47 ± 1.91, (ranging between 0 and 8). The ATLAS values in the R group (A_R) were not significantly increased versus the NR group (A_NR) (*p* > 0.05) (Figure 2).

**Figure 3:**
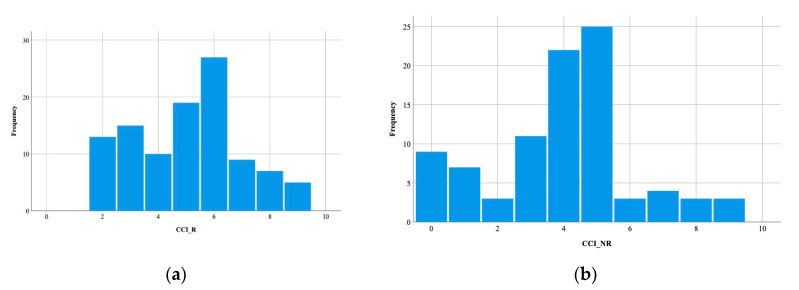
Histogram of values of Charlson Comorbidity Index: (**a**) in the recurrent group; (**b**) in the non-recurrent group. CCI_R—Charlson Comorbidity Index in the in the recurrent group, CCI_NR—Charlson Comorbidity Index in the non-recurrent group.

The analysis of the CCI values of the patients with CDI, during the studied period, revealed a value of 4.55 ± 2.128 for CCI, (ranging between 0 and 9). The CCI values in the R group (CCI_R) were significantly increased versus the NR group (CCI_NR) (5.07 ± 3.967 vs. 1.94 ± 2.187, *p* < 0.001 (Figure 3a,b).

**Figure 4:**
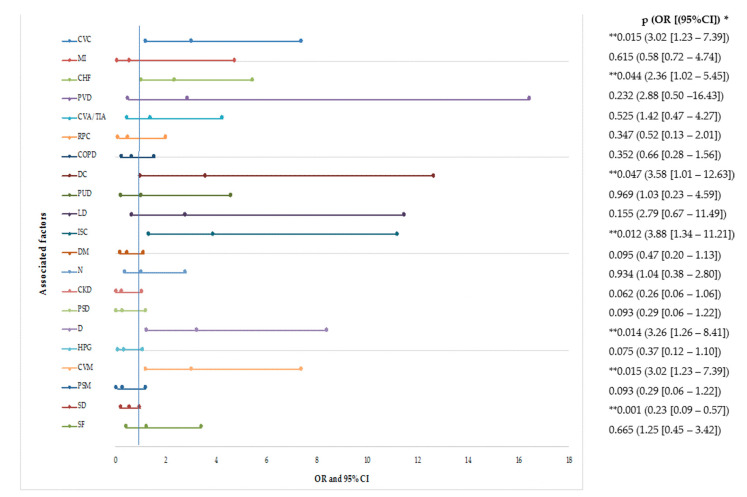
Forest plots of the associated factors with recurrent CDI (logistic regression analysis). * Logistic regression analysis, ** *p* values < 0.05, OR—odd ratio, CI—Confidence interval. Legend: CVC—cardiovascular disease comorbidities, MI—myocardial infarction, CHF—congestive heart failure, PVD—peripheral vascular disease, CVA—cerebrovascular accident, TIA—transient ischemic attack, RPC—respiratory comorbidities, COPD—chronic obstructive pulmonary diseases, DC—digestive comorbidities, PUD—peptic ulcer disease, LD—liver disease, ISC—immunosuppressive comorbidities, DM—diabetes mellitus, N—neoplasm (solid tumor, leukemia, lymphoma), CKD—chronic kidney disease, PSD—psychiatric diseases, D—dementia, HPG—hemiplegia or paraplegia, M—medication, CVM—cardiovascular medication, PSM—psychiatric medication, SD—symbiotic drug, SF—severity form, R—Recurrent, NR—Non-recurrent, OR—odds ratios, CI—confidence intervals, *p* values—statistical significance.

The statistical analysis identified cardiovascular disease comorbidities (CVC), digestive comorbidities (DC), dementia (D), immunosuppressive comorbidities (ISC), and cardiovascular medications (CVM) as risk factors for recurrence of CDI, while the use of a symbiotic drug (SD) is a protective factor against it (Figure 4).

## 4. Discussion

### 4.1. Associated Factors with Recurrent CDI

Recurrence is a complication of CDI that exposes the patient to be hospitalized again; it involves aggravation of associated pathologies and increased costs for the health care system. Often, diagnosing these patients can be problematic and tiresome (for both the patients and the clinicians). In the current study, one out of two patients with CDI developed at least one recurrence in the 3 months following the first episode. The recurrence rate obtained (53.84%) was higher than that found in the medical literature. About 15–47.2% of patients initially treated for CDI developed, in the following weeks, at least one new episode of CDI [2,19]. The high relapse rate obtained in the study could be explained by the use of metronidazole as the first line of treatment, according to the national CDI treatment guidelines. International treatment guidelines for CDI recommend vancomycin or fidaxomicin as first-line drugs rather than Metronidazole [20]. The number of recurrence episodes because of CDI, obtained in the current study (60.95%–single recurrence, 39.05%–multiple episodes), is almost identical to the one reported by the medical literature. Marsh et al., in a 2012 study on 82 patients with recurrence of CDI admitted to the University of Pittsburgh Medical Center Presbyterian, Pennsylvania, USA, reported an incidence of 66.41% for a single episode, and 36.58% for multiple recurrences [21].

#### 4.1.1. Demographic Factors

The mean age of patients with CDI was around 69 years, without a statistically significant difference between the two followed groups. In a study conducted by us in 2020, on 877 patients with CDI, the age group 55–74 was most affected by CDI [22]. Most of the patients with CDI were aged between 60 and 69 years—the average age at which patients experience the aging phenomenon associated with immunological senescence and the modification of gut microbial composition [23]. Decreased estrogen synthesis induces a decline in the Th_2_-type immune response, a response that is more effective against extracellular pathogens and toxins, being mediated by interleukins 4 and 5 [24,25]. Human microbiota plays a crucial role in the emergence of CDI. With age, the gut ecosystem undergoes major transformations [26,27,28]. Firmicutes and Bacteroidetes counted 99% of bacteria from human microbiota, and it is well known that their ratio decreases with age [27]. Moreover, *Bifidobacterium* and *Lactobacillus* decline with increasing age [29,30]. Wei Y. et al. showed (in an in vitro study in 2018) that the presence of bifidobacterial strains determined the acidification of the environment, inhibition of growth, and reduction of the CD toxin strains synthesis [31].

A higher number of patients without recurrences from rural areas (67.77%) was identified, but the differences obtained in the present study were not statistically significant. Surprisingly, the medical literature has a contradictory opinion regarding this issue. The urban population has much easier access to healthcare services compared to patients in rural areas; exposure to animals (cattle, pigs) and a contaminated environment is often more common in rural areas [22,32,33], leading to higher exposure to spores of CD. In a study led by Redding L. et al. (from the University of Pennsylvania Health System, Philadelphia, PA, USA, in 2020) on a group of 232 patients with CDI, the authors claimed that pet owners are protected against recurrences of CDI due to modification of microbiota secondary to cohabitation with the animals [34].

Although different hormone levels can influence the gut microbiome and the constellation of associated diseases, in this study, gender differences were not identified as a risk factor for recurrence of CDI [35].

#### 4.1.2. Clinical Factors

The ATLAS score is used for the stratification of patients with CDI according to the severity of the disease. A patient with severe disease typically has a weakened immune system, altered gut microbiota, or has other associated severe pathologies. In this study, although the values of the ATLAS score were higher in the group with recurrent CDI (4.59 ± 1.69), the differences were not statistically significant. However, the current medical literature is contradictory, thus, in a study, Jacobson S.M. et al. argued, in 2015, based on data collected from 245 adult patients with CDI, that the ATLAS score could not predict CDI recurrence [36]. In a study by Krafl D. et al. published in 2019, using data collected from 144 patients with CDI, at the University Hospital of Vienna, the authors claimed that the ATLAS score is not associated with recurrence of CDI in the 90 days following the first episode, but the level of albumin (a component of the ATLAS score) is positively correlated with the recurrence rate [3]. Moreover, the ATLAS score values influence the treatment decision, and the hypervirulent strains of CD are responsible for severe forms of the disease; thus, increased values of the ATLAS score, and until now, the medical literature recognized that the hypervirulent strains of CD, or treatment with fidaxomicin or vancomycin, may influence CDI recurrence rates [1,6,7,8,37,38].

Patients with comorbidities are more frequently exposed to healthcare services, with the secondary risk of CD colonization or the use of medications. Recurrence of CDI occurs secondary to an inefficient immune response or intestinal dysbiosis. CCI includes several chronic diseases that could modify both microbiota and immune system response, leading to CDI recurrence. This study found significantly higher CCI values in the R group (5.07 ± 3.967) compared to the NR group for patients with CDI. The results obtained are consistent with the medical literature. Aguilar-Olivos N.E. et al., in a 2016 study performed on 167 Mexican patients diagnosed with CDI, claimed higher CCI values in the group that presented recurrences [39].

Worldwide, cardiovascular disease is the leading cause of death, amounting to 17.9 million deaths annually [40]. The relation between intestinal dysbiosis and heart disease is intensely debated. Intestinal dysbiosis can favor metabolic syndrome via the parasympathetic nervous system, secondary to increased production of acetate by gut microbiota [41]. Substances synthesized following the activity of the gut microbiome, such as trimethylamine N-oxides, short-chain fatty acids, and secondary bile acids can intervene on the frequency of cardiovascular events [42,43,44]. In the present study, it was found that the risk for recurrent CDI is 3.02 times higher in patients with cardiovascular comorbidities or cardiovascular treatment (OR = 3.02, 95% CI = 1.23–7.39, *p* = 0.015), but not individually for myocardial infarction, peripheral vascular disease, cerebrovascular accident, or transient ischemic attack. Patients with congestive heart failure have a 2.36-fold increased risk of CDI recurrence (OR = 2.36, 95% CI = 1.02–5.45, *p* = 0.044). In a study published in 2016 by Mamic P. et al., in 5,851,582 patients with heart failure from the United States, it was revealed that this type of comorbidity was more frequently associated with CDI (OR = 1.13, 95% CI = 1.10–1.16) [45].

The link between human gut microbiota and digestive diseases is intensely studied in the medical literature. The presence of *Helicobacter pylori* (HP) can induce gastric inflammation via modification of gut microbiome, and concentration of antimicrobial peptides from the gastrointestinal tract can modulate host immune response against HP [46]. Moreover, the increase of gastric pH favors the survival of CD strains. Recent studies claim that intestinal microbiota is linked with the presence of chronic intestinal inflammation and, therefore, with inflammatory bowel diseases [47]. In time, the last one will be responsible for psychic changes, which will require specific medication [48,49]. However, medical literature is contradictory regarding the effect of psychiatric medication on inducing intestinal dysbiosis. Inflammatory bowel disease is manifested by diarrheal syndrome, frequently treated with antibiotics, which exacerbates gut dysbiosis [47]. *Giardia lamblia*, a parasite highly frequent in the Romania population [50], can induce dysbiosis and post-infectious irritable bowel syndrome, mediated by the toll-like receptor 4 pathway and overproduction of the interleukin-1 beta [51]. Chronic liver disease requires long term hospitalization, antibiotics (usually) to prevent hepatic encephalopathy, and proton pump inhibitors to reduce the future complications of cirrhosis [52]. In the present study, we identified that patients with digestive diseases have a 3.58 times higher risk of recurrent CDI, compared to patients without this pathology (OR = 3.58, 95% CI = 1.01–12.63, *p* = 0.047). Individually, peptic ulcer disease and liver disease have not been identified as being responsible for the increased risk of recurrence of CDI. The medical literature is contradictory on this topic. A retrospective study, published in 2019, and conducted by Dharbhamulla N. et al. (at Cooper University Hospital, USA) on a group of 435 patients with recurrent CDI, claims that colectomy is a risk factor for recurrence (OR = 1.023, 95% CI = 1.009–1.037, *p* < 0.05), but the use of proton pump inhibitors is not [53]. Appaneal H.J. et al., in a national study published in 2019 conducted on 974 cases, claim that antibiotics, proton pump inhibitors, and biliary tract disease increase the risk of recurrence of CDI [54].

Immunosuppression leads to higher consumption of antibiotics, increases the addressability to health services, and induces the breakdown of natural barriers against CD. A wide range of diseases and drug treatments can induce a weakened immune system. Diabetes and cancer are two chronic diseases responsible for immunosuppression, presenting an alarming increase in recent years. Our research identified that immunosuppression increases by 3.88 times the risk of recurrence of CDI (OR = 3.88, 95% CI = 1.34–11.21, *p* = 0.012). However, a direct relationship between recurrences of CDI and diabetes or cancer was not identified. Avni T. et al., in a study published in 2020, conducted on 573 immunocompromised patients, shows the same risk for the recurrences of CDI (OR 2.7, 95% CI 1.6–5) [55].

#### 4.1.3. Environmental Conditions

Environmental conditions, stress, vitamin deficiency, endocrine disorders, structural injury of the brain, physical factors, and other associated pathologies are factors that, over time, can lead to an increase in the frequency of dementia with aging [49,56]. Often, people with dementia, suffering from memory loss, are left by their families in care centers or are immobilized in bed, being more frequently exposed to health system deficiencies/infections, as well as the use of antibiotics or other medications that could induce dysbiosis. The relationship between gut and brain, named gut–brain axis, mediated by the vagus nerve, neurotransmitter, neurohormones, immune system, is well established. Depression is based on chronic inflammation of the brain, and, via cytokines, responsible for changing gut permeability and microbiome composition. Increased gut permeability leads to the augmentation of the synthesis of the pro-inflammatory cytokine and secondary to inflammation [57]. Moreover, the medication used in the treatment of mental illness can bring changes to the gut microbiome [57,58]. In the study, dementia increases the risk of relapse of CDI by 3.26 times (OR = 3.26, 95% CI = 1.26–8.41, *p* = 0.014). Until now, the relationship between dementia and recurrence of CDI has not been established.

The effect of various symbiotic on the gut microbiota is well known to date. Due to the great diversity of symbiotics on the market, so far there is no unitary idea regarding the use of these products in the treatment of CDI. Moreover, some studies claim the idea of sepsis with bacterial strains from symbiotics, secondary to the administration of these drugs to patients with CDI [59,60,61]. The present study claims that 3-months use of four bacterial strains with symbiotic drugs have a protective effect against the recurrence of CDI (OR = 0.23, 95% CI = 0.09–0.57, *p* = 0.001). Deshpande et al., in a study published in 2013 on solid organ transplant patients, claimed that Lactobacillus administration can reduce the incidence of CDI recurrences 3-fold (OR = 0.29, 95% CI = 0.1–0.8, *p* = 0.01), and bacteremia was not reported [62].

## 5. Conclusions

The results of the present study sustain that recurrence of CDI appears in one of two patients hospitalized for this pathology. Patients aged 60–69 years, or those with higher CCI, are prone to CDI recurrence. The presence of cardiovascular pathology increases the risk of future CDI episodes in the 3 months by 2.8 times, digestive disease by 5 times, dementia by 2 times, and immunosuppression by 2.5 times. The symbiotic drug, for 3 months, has the effect of protecting patients from recurrences.

## Figures and Tables

**Table 1:** Distribution of the patients according to the age groups.

Age group (Years)	R	NR	*p*
<50, M, SD (N)	42.50 ± 6.73 (14)	38.15 ± 10.23 (13)	0.200
50–59, M, SD (N)	56.00 ± 2.27 (23)	57.00 ± 0.0001(6)	0.297
60–69, M, SD (N)	66.92 ± 1.44 (13)	64.14 ± 1.06 (7)	<0.001
70–79, M, SD (N)	75.70 ± 3.49 (30)	76.41 ± 2.96 (54)	0.326
>80, M, SD (N)	84.16 ± 2.15 (25)	85.10 ± 2.18 (10)	0.253

M—mean, SD—standard deviation, *p* values—statistical significance, CDI—*Clostridioides difficile* infection, N—number of cases, R—recurrent, NR—non-recurrent.
